# The role of primary dental care practitioners in the long-term management of patients treated for head and neck cancer

**DOI:** 10.1038/s41415-022-5148-z

**Published:** 2022-11-11

**Authors:** Harpoonam Kalsi, Lorna K. McCaul, Jose M. Rodriguez

**Affiliations:** 4141573680001 Consultant in Restorative Dentistry, Guy´s and St Thomas´ NHS Foundation Trust, 26th Floor, Tower Wing, Great Maze Pond, London, SE1 9RT, UK; 4141573680002 Consultant and Honorary Clinical Senior Lecturer in Restorative Dentistry, Department of Restorative Dentistry, Glasgow Dental Hospital and School, NHS Greater Glasgow and Clyde, Glasgow, Scotland, UK; 4141573680003 Consultant in Restorative Dentistry and Honorary Clinical Senior Lecturer, Faculty of Dentistry, Oral and Craniofacial Sciences, King´s College London, 26th Floor, Tower Wing, Great Maze Pond, London, SE1 9RT, UK

## Abstract

Patients treated for head and neck cancer may be susceptible to a higher incidence of dental disease due to long-term sequelae of treatment for head and neck cancer. Most patients with head and neck cancer are discharged from a hospital environment and responsibility for long-term dental care is transferred back from the restorative dentistry team to the dentist and dental care professionals in primary care. Treatment of these patients should be undertaken in a supportive environment, taking into account the physical and psychological repercussions of previous treatment. With the exception of some surgical procedures, routine dental care is not contraindicated in patients after head and neck cancer treatment and it is expected that the dentist and dental care professionals will be responsible for long-term routine dental treatment. Primary dental care practitioners should be aware of the process to refer patients back to the head and neck cancer multidisciplinary team if they note a suspicious change during their routine clinical examinations. Referral to a restorative dentistry consultant for planning and carrying out complex items of care may sometimes be necessary, but patients should always remain under the long-term care of their primary dental care practitioner.

## Key points

- Following discharge from the specialist cancer centre after head and neck cancer treatment has completed, patients should receive routine ongoing dental care within the primary care setting, supported by restorative dentistry services when appropriate.
- Surgery and radiotherapy have long-term side effects which may predispose patients to greater incidence of primary dental disease; prevention is key to minimise dental complications.
- Osteoradionecrosis of the jaw is a risk of surgical procedures in sites which have received radiotherapy. Advice should be sought from the multidisciplinary team if considering surgical procedures.

## Introduction

There are in excess of 12,400 new head and neck cancer (HNC) diagnoses in England and Wales per year, with increased incidence in younger populations due to association with human papillomavirus 16.^1^ Primary dental care (PDC) teams including community dental services, public dental services, general dental practitioners, and dental care professionals, play a crucial role in early detection, monitoring and long-term dental management of patients suspected of, or treated for, HNC. As secondary care services are under pressure to recover clinical activity post COVID-19, patients treated for HNC are expected to be discharged from hospital care once their treatment has completed. The PDC practitioner plays a crucial role in the long-term management of patients treated for HNC after they are discharged from the multidisciplinary team (MDT) and from the care of the restorative dentist. Following cancer treatment, patients can be left with a wide range of morbidities, from slight speech difficulties to severely altered oral anatomy with a dry mouth, depending on the type of treatment they have received, which may involve surgery, radiotherapy, chemotherapy, or more often than not, a combination of modalities. This paper discusses the role of the PDC practitioner in long-term dental management strategies for patients treated for HNC.

## Overall strategies

While planning and undergoing active HNC treatment, patients will be under the care of the restorative dentistry team as part of the HNC MDT. Oral and dental treatment strategies at that time are carried out with reference to guidance and standards published by the National Institute for Health and Care Excellence (NICE), the British Association of Head and Neck Oncologists, Restorative Dentistry-UK, ENT UK and the Royal College of Surgeons of England. On discharge to the PDC practitioner, there are understandable anxieties for dentists in managing patients who have been treated for HNC. Patients may present with long-term complications of treatment which may affect provision of routine dental treatment. Surgery results in altered anatomy and scarring and radiotherapy can cause osteoradionecrosis risk as well as xerostomia, trismus, dysphagia and loss of taste.^2^ Alongside the physical side effects, patients can be left with psychosocial problems related to their diagnosis and treatment and reduced quality of life, which can all impact on the ability to provide dental care.^3^ Patients treated for HNC may also develop long-term, potentially preventable, complications, such as caries and periodontal diseases, which, if left uncontrolled, can have devastating effects on a patient's dentition and overall quality of life.

The care for patients with HNC is coordinated through local MDT clinics which involve all members of the team involved in cancer care. NICE guidelines specify that a consultant in restorative dentistry should attend the MDT in order to assess patients' dental needs before treatment, support patients during treatment and rehabilitate patients after cancer treatment when necessary.^4^ After surgical or non-surgical cancer treatment has been completed, patients are normally followed-up by ear, nose and throat or oral and maxillofacial surgeons for five years. Following surgery, patients will be discharged by the restorative dentist to the care of their primary dental care practitioner with a written plan for long-term monitoring and management of dental needs once specialist oral rehabilitation is complete. Following radiotherapy, patients may be discharged to the care of their PDC practitioner within weeks of completing treatment if they are able to maintain oral hygiene comfortably and are able to self-manage caries risk within their diet. Once discharged, the PDC practitioner can follow the *Delivering better oral health *toolkit^5^ in order to determine appropriate preventative and maintenance protocols for their patients.

## Long-term monitoring

During the first six months to one year post treatment, HNC patients will be followed-up on a regular basis by their head and neck surgical team but they should continue to attend their routine dental appointments during this period. It is important for the PDC practitioner to document a thorough baseline examination of a patient after they are discharged from cancer treatment and any changes to the baseline examination should be investigated further. The PDC practitioner should remain vigilant during their routine examinations and carry out extra-oral and intra-oral examinations, documenting any changes, including the absence of any change. The extra-oral examination should include assessing the lymph nodes to compare it to the assessment at baseline. Neck dissections are commonly undertaken alongside surgical tumour excision, which can leave the patient with scarred submandibular and neck regions, and which can be difficult to assess clinically. The focus of the examination should be on any changes that may have occurred since their previous visit. The intra-oral examination should assess mucosal changes and compare these to the baseline assessment. Additionally, pre-op baseline photographs are a good tool to assess for changes. Patients post treatment may present with altered anatomy due to surgery which would have an impact on the clinical examination. A good set of baseline intra-oral and extra-oral photographs is useful to compare any changes at future examinations. Patients who have been treated with radiotherapy may present with difficulties swallowing due to a dry mouth, so it is important to document swallowing abilities at baseline to compare any possible changes. Any concern should prompt a referral to the appropriate member of the MDT.

## Risk factor mitigation

Risk factors for cancer have been discussed in another paper in this themed issue. The PDC practitioner should be closely involved in risk factor modification, including smoking cessation advice using NICE guidelines^6^ and counselling patients in reducing alcohol intake. An updated medical history at each visit is important to help pick up changes in behaviour which could be a risk for HNC and seek referral from the appropriate services/general practitioner where appropriate.

## The role of dental disease prevention strategies

Prevention of dental disease during and immediately after HNC treatment can be challenging and will be managed within the MDT. Following discharge to primary care, preventative regimes are the most important factor to ensure that risk of primary dental disease is reduced as future dental treatment may be challenging to deliver in some patients due to long-term complications. The dentist, hygienist, therapist and dental nurse are key in offering preventative dental advice and to engage patients in their long-term dental care following published guidelines.^5^ Clinicians and dental care professionals should offer preventative advice in a sensitive way considering that their patient may have long-term physical and psychological sequelae of treatment for HNC. Patients should be treated with sensitivity regarding these factors and we should aim to offer 'support' and education rather than 'instructions'. The initial findings from the OraRad study highlighted the importance of oral hygiene support and regular follow-up care in reducing the risk of substantial tooth loss post radiotherapy treatment.^7^ Patients should be aware of the risks of sucrose- and glucose-containing oral nutritional supplements and other sources of fermentable carbohydrate as the primary cause of caries. Discomfort from persistent mucositis may reduce tolerance to topically applied preventive agents, such as mouthwashes and toothpaste. The prevalence of radiotherapy-related dental caries has been shown to be over 25% and is thought to be partly related to hyposalivation and changes to the composition of the saliva which reduces its protective effect.^8^ Long-term dental disease prevention strategies are outlined in Table 1.

**Table 1:** Long-term caries prevention strategies for patients with head and neck cancer after discharge to primary care

**Stage**	**Toothbrushing**	**Toothpaste**	**Inter-dental cleaning**	**Mouthwash**	**Other measures**
After cancer treatment	Resume twice daily toothbrushing with a manual or electric toothbrush as soon as acute symptoms of treatment have subsided. In cases where patients have post-operative trismus, recommend the use of smaller toothbrushes with regular strength bristles (eg single-tufted brush)	Continue/ resume 5,000 ppm fluoridated toothpaste as soon as any mucositis symptoms have resolved. In patients with a high caries risk or those with reduced salivary flow, prescribe 2,800 ppm (<16 yo) or 5,000 ppm (>16 yo) fluoridated toothpaste. On patients with xerostomia as a result of radiotherapy, use CPP-ACP Tooth Mousse (GC Europe) by gently can be applied to the teeth twice daily	Encourage once daily inter-dental cleaning with inter-dental brushes or floss	Fluoridated mouthwash at other times than toothbrushing	Advise to spit but not rinse toothpaste. For patients on long-term Oral Nutritional Supplements, dietary counselling is needed to mitigate the risks. Encourage patients to brush their teeth or rinse with a fluoridated mouthwash before ONS. Consider use of upper and lower soft splints to be worn at night which can be used as fluoride/CPP-ACP Tooth Mousse (GC Europe) reservoirs

### Oral hygiene

Patients treated for HNC are at higher risk of preventable dental disease due to a number of factors. Patients treated for surgery may present with altered anatomy which may compromise effective oral hygiene regimes, as patients may have difficulty accessing their teeth due to trismus, post-surgical microstomia, reduced mobility of the tongue, or tethered flaps causing tight buccal mucosa. Additionally, patients with palatal defects may find it difficult to brush their teeth, rinse their mouth, or use mouthwashes due to choking with nasal escape of fluids. Patients should be counselled on how to achieve good oral hygiene levels, including alternatives to standard approaches, such as using small toothbrushes to access tight areas and using water flossers when it is not possible to use floss or inter-dental brushes. A single-tufted toothbrush can be useful for accessing palatal or lingual tooth surfaces if trismus is present. If a patient with a palatal defect struggles to clean effectively, they can be counselled to brush and rinse with the obturator *in situ* and then remove the obturator to clean the palatal surfaces with minimum amount of liquids in their mouth. An alternative is to liaise with the restorative dentist to fabricate a cover plate with open palatal margins which can be used during brushing and can seal the airway from the oral cavity. Denture hygiene also needs to be emphasised.

### Dietary counselling

There is evidence that patients treated for HNC have sub-optimal nutrition which has systemic implications.^9^ The role of the specialist dietitian in supporting patients with HNC is essential to ensure that patients receive adequate nutrition before, during and after treatment and this has been discussed in another paper in this themed issue. Some patients may have to remain on oral nutritional supplements (ONS), such as Fortisip and Ensure, long term. These supplements are carbohydrate-rich and can be highly cariogenic, with some patients consuming these supplements 6-8 times a day over a protracted period of time. This effect can be worsened during radiotherapy when they are at a high risk of caries due to hyposalivation and have difficulties achieving adequate oral hygiene due to mucositis and trismus. During this period, the need to maintain nutritional intake takes precedence and patients are counselled by the restorative dentistry team before commencing treatment in how to mitigate caries risk of frequent carbohydrate consumption. Patients are reviewed within weeks of completion of treatment and members of the MDT will liaise with the dietitians regarding cessation of ONS, if possible. Following discharge to the PDC practitioner, routine preventative advice should be given, including use of a fluoridated mouthwash after foods, effective plaque removal, reduction of consumption of sugary food and beverages outside of main meals and continued use of high concentration fluoride toothpaste. Toothpastes containing casein phosphopeptide-amorphous calcium phosphate (CPP-ACP) may be a useful adjunct in preventing caries in patients with xerostomia who cannot cease ONS use.

### Fluoride regimes

Patients with reduced mouth opening, post-treatment xerostomia, or on highly cariogenic diets, are at high risk of dental caries, so a more intensive fluoride regime must be considered. High concentration fluoridated toothpaste can be prescribed with the advice that the patient does not rinse their mouth after brushing their teeth, with use of a fluoridated mouthwash at other times than toothbrushing. Topical adjunctive, such as CPP-ACP (Tooth Mousse - GC Europe) can also be considered but they are not available under NHS contracts. Patients can be instructed on the regular, nightly use of custom fluoride trays to also help prevent dental caries, which has shown to be effective even where strict dietary control cannot be implemented.^10^

## Restorative dentistry procedures in primary care

A common misconception is that patients treated for HNC must receive all future dental care in a hospital setting. This is not appropriate as there are no additional contra-indications for non-surgical restorative treatment by the PDC, routine care is best provided closer to homereducing unnecessary visits to hospital, with less travel time and associated costs. Additionally, acute trustsdo not have the capacity for long-term treatment of this large cohort of patients. Following their dental rehabilitation, patients with HNC are routinely discharged back to the care of the PDC practitioner who is responsible for providing routine dental care. There are no additional contraindications for non-surgical restorative treatment in patients with HNC, so operative treatment should be the same as a patient who has not had cancer. If complex dental treatment involving dental implants has been provided, suggested maintenance plans are also usually provided and if any issues occur which cannot be managed in primary care, the patient can be referred back to the restorative dentistry department for management.

Secondary to changes in the blood vessels, such as endarteritis obliterans,^11^ patients who have undergone radiotherapy have been shown to be at increased risk of periodontal breakdown due to reduced vascularity of the oral tissues, therefore oral hygiene optimisation is essential in prevention of disease.^12^ Periodontal treatment should be carried out by a dentist or a dental care professional. Guidelines for the management of periodontal disease have been published by the British Society of Periodontology and these are applicable to patients treated for HNC. Recall protocols may need to be more regular for this cohort of patients as they will need closer monitoring including assessment of dentures to ensure they are not causing trauma to the soft tissues.

Orthograde root canal treatments can be provided without any contraindications and are sometimes provided on teeth which are unrestorable to avoid a high-risk extraction due to a risk of osteoradionecrosis (ORN) of the jaw. Guidance from the restorative department may be sought if there is doubt regarding restorability and/or ORN risk with extraction. Prosthodontics and conservative dentistry can be carried out safely but the emphasis should be on keeping restoration margins supra-gingival where possible and to ensure all restorations are fully cleansable. Primary care dentistry should be provided by dentists and dental care professionals. Patients can be referred to a restorative dentistry consultant for advice if required and also for provision of complex restorative care, such as obturators, complex dentures or implant-retained prostheses.

## Planned oral surgery procedures

Surgery, such as implant placement or dental extraction, in a previously irradiated site carries the risk of ORN of the jaws. ORN is described as exposed irradiated bone that fails to heal over a period of three months without evidence of persisting or recurrent tumour.^13^ ORN risk is associated with any invasive surgical procedure causing trauma to the bone in a previously irradiated site, or can occasionally arise without any trauma. When in doubt, it is better to refer these patients to a hospital environment where the risks can be properly assessed. If a patient has had radiotherapy, the radiation fields can be obtained in order to assess the specific dose to the specific site and assess the safety of surgery. In the past, hyperbaric oxygen treatment has been advocated as an intervention to reduce the risk of ORN following post-radiotherapy extractions and dental implant placement in irradiated bone, however, a randomised controlled trial undertaken by Shaw *et al*., indicated its limited effectiveness^14^ which has led to research into other possible types of treatment, such as the prophylactic use of pentoxyfylline and tocopherol.^15^^,^^16^ Surgery in general practice should be avoided in patients who have had a post-operative reconstruction with a free-flap as there is a risk of severing the anastomosis which would result in necrosis of the flap. Surgery in close proximity to flaps should be carried out in a hospital setting.

## Suspicion of osteoradionecrosis

If a patient who has a previous history of radiotherapy treatment presents with an area of suspected ORN, they should be referred to secondary care so the diagnosis can be confirmed and managed. This may range from simple conservative management to surgical debridement or medical management using pentoxifylline, tocopherol and clodronate. The latter treatment is showing promising results^17^ but is still not considered standard treatment.

## Conclusion

Once patients have completed treatment with the restorative dentistry team and no longer require specialist care, they will be referred to general dental practice with a written long-term plan. For patients who do not have a PDC practitioner, the restorative dentistry team should have arrangements with the local community dental service (England, Wales, Northern Ireland) or public dental service (Scotland) for long-term care, where patients can be managed by special care dentistry teams in those services and can be referred to restorative dentistry if any oral rehabilitation issues arise.

The PDC practitioner has an essential role in the long-term supporting and monitoring of patients following discharge after treatment for HNC and as such, are viewed as an integral part of the extended clinical team. Patients should remain under long-term care with a PDC practitioner, with appropriate referral to the restorative dentistry consultant for queries in relation to restorative dental care, or referral to the HNC MDT if there is a suspicion of recurrence when a new primary is suspected or when complications arise. Patients should be able to receive dental care close to where they live so the PDC practitioner plays a crucial role in delivering ongoing care.
